# Addressing Child and Adolescent Mental Health Problems in the Community. Evaluation of a Consultation and Advise Team for Assessment, Support and Referral

**DOI:** 10.5334/ijic.8584

**Published:** 2024-10-24

**Authors:** Mariëtte H. H. Hoogsteder, Sumayah Vandenbussche, Marieke Zwaanswijk

**Affiliations:** 1 Amsterdam UMC location Vrije Universiteit Amsterdam, Department Public and Occupational Health, De Boelelaan 1117, Amsterdam, The Netherlands; 2 Amsterdam Public Health, Quality of Care, Amsterdam, The Netherlands; 3 Trimbos Institute –for Mental Health, Utrecht, The Netherlands

**Keywords:** mental health problems, preventive youth health care, primary care, integrated care, interprofessional collaboration

## Abstract

**Introduction:**

Youths with mental health problems are often not identified in primary healthcare, which may prevent or delay appropriate support. In the Netherlands, a Consultation and Advise expert team (CandA team) was implemented to support general practitioners (GPs), youth professionals and youths with mental health problems. This study investigates the team’s scope, activities, stakeholders’ and users’ experiences.

**Method:**

Interviews and focus groups with policymakers, healthcare professionals, parents and youths were analysed using ATLAS.ti. Demographics and mental health problems of 706 youths (0–18 years) consulting the CandA team, type of healthcare providers consulting the team, and type of care provided by the team (2015–2017) were analysed, using descriptive statistics and Chi-square tests.

**Results:**

Youths consulted the CandA team for ‘other behavioural/psychological complaints’ (41%); irritable/angry behaviour (14%); anxious/nervous behaviour (10%); overactivity (8%); feeling down/depressed (6%). CandA team services were used by GPs, youth counsellors, and youth physicians/nurses. Most stakeholders were positive about the team’s services.

**Conclusion:**

The CandA team seems an adequate form of integrated assessment and support for youth mental health problems in the community. The team’s composition, expertise and positioning are success factors. Cooperation with schools could be improved. Quantitative evaluation is needed to investigate effects of the team and adequacy of referrals.

## Introduction

### Background

Worldwide, mental health disorders are prevalent in 8–14% of children and adolescents [1]. In the Netherlands, 11–43% of the young population experiences mental health problems, including emotional, behavioural and psychosocial problems [2,3,4,5]. Prevalence rates vary by gender and age groups [2,3,4,6,7]. Dutch children’s and adolescents’ mental health problems have increased in previous decades [5,7]. Utilization of youth mental health care increased as well, both in the Netherlands as elsewhere [8,9,10,11]. However, there is still a considerable unmet service need for youth mental health problems [5,8,11,12]. Mental health care therefore faces the dilemma of matching its resources with increasing demands.

Most common mental health problems are depression (anxious or withdrawn), psychosomatic problems and rule-breaking problems [7]. Child and adolescent mental health in the Dutch general population has deteriorated during the first year of the COVID-19 pandemic, and although it has improved since then, it has not returned to pre-pandemic levels [3,13]. Mental health problems can negatively affect children’s current and future functioning. Early identification and adequate treatment are important to prevent worsening of problems, leading to increased use of care and higher costs for society [14].

Previous research indicated that many children and adolescents with mental health problems are not identified by primary health care providers [4,15,16]. Although not all of them will need additional care, the limited identification of mental health problems may prevent or delay the receipt of appropriate care for the ones who do [4,5]. On the other hand, some youngsters may unnecessarily or too easily be referred to specialized care, because of lack of time or expertise of general practitioners (GPs) and limited collaboration between health care professionals from different domains in youth health care [17,18,19,20].

### Professionals involved in Dutch youth mental health care

In the Netherlands, several types of professionals are involved in the care and support for youth with mental health problems, who are all children and adolescents from 0 to 18 years of age. First, Dutch GPs function as gatekeepers with regard to the physical and mental health of children and referral to specialized health care. Almost all inhabitants are registered in a general practice, which is close to the community. GPs are usually the first health care providers consulted when parents have concerns about their child’s physical or mental health [4]. Most Dutch children and adolescents consult their GP at least once a year [21]. Some general practices include a physician’s assistant for mental health.

Secondly, Youth health care (YHC) physicians are crucial in the early detection of mental health problems, as they have regular check-ups with all children. YHC offers free of charge: (preventive) information and education, vaccinations, monitoring and screening physical and mental health, identification of care needs and preventive support to children and their families from birth to the age of 18 years [22,23]. YHC physicians all have specialised training and expertise in social medicine and the development of children and adolescents [19,24]. Like GPs, YHC physicians have the authority to refer children to youth (mental health) care [22]. When problems are identified, YHC physicians decide whether there is any need for advice, further assessments by YHC or referral. Referrals matching the needs of youth are an essential component of YHC services.

Other professionals involved in providing Dutch youth mental health care are professionals of social district teams. Access to these services is the responsibility of municipalities, which can offer the services themselves or outsource them to public or private welfare organisations [25].

Finally, social workers, paediatricians, professionals from primary and specialized youth mental health care and professionals from child protection services provide youth mental health care. These services are only accessible after referral.

### Dutch youth mental health care reform

Before 2015, Dutch services for youth were fragmented, with various financial flows, and little interprofessional collaboration. Improving the integration of services and the adaptation to local needs was needed. This led to a transition of youth services from national and provincial to local and regional policy.

After the introduction of a new Youth Act (2015), the 355 Dutch municipalities became responsible for all welfare, support and mental health care for children, adolescents, and families in need of help. All administrative and financial responsibilities were transferred to the local level [17,26]. The main aim of this Act was to decrease the number of youths in specialized care and increase preventive, timely and customized care for youth with mental health problems [17].

After 2015, policy makers and professionals have been organizing new ways of working [19,27,28]. One example was implemented in the Dutch region ‘Gooi en Vechtstreek’, a collaboration of seven municipalities in the centre of the country, with about 240,000 inhabitants, among which nearly 60,000 youth between 0 and 18 years old. In 2015, an independent expert team (the Consultation and Advise team for youth mental health, CandA team) was implemented in this region to support professionals, mainly GPs, with the identification of child and adolescent mental health problems and the provision of appropriate care.

Although YHC physicians can play an important role in supporting GPs and other referrers in the early detection and treatment of children and adolescents with mental health problems, initiatives to support GPs in the care for youths with mental health problems seldom involve YHC [29]. Collaboration between GPs and YHC physicians is generally suboptimal with respect to knowledge of each other’s competencies and tasks, trust, information exchange and support from within organisations or municipalities [30,31]. The CandA team, including two YHC physicians, was aimed to improve care for youth with mental health problems, according to an integrated care model [20].

### The development of the CandA team

Preceding the implementation of the Youth Act, local authorities in the Dutch region ‘Gooi en Vechtstreek’ started to collaborate with GPs to keep track of their referral practices and limit the costs for the municipality, as they – instead of insurance companies – would become financially responsible for all youth mental health care. Most GPs considered themselves to have insufficient time and expertise to diagnose and treat youth mental health problems. Professionals from youth care and welfare agencies indicated they needed extra expertise in child and adolescent mental health. Because of their knowledge, expertise and responsibilities, YHC physicians considered themselves as key players in the future landscape of youth health care [32]. This resulted in a pilot of the CandA team in 2014, consisting of two YHC physicians with extra training in mental health, and a clinical psychologist specialized in children and adolescents. In the last three months of 2014, this team intensively collaborated with four GP’s. All youth cases and the working procedure were reviewed by mental health professionals from five regional mental health organisations. All professionals involved in the pilot were enthusiastic. As a result, the CandA team was implemented in seven municipalities as of 1 January 2015. A remedial educationalist, a child and adolescent psychiatrist or a paediatrician could be consulted if necessary.

All GP’s in the region were informed in a letter about the CandA team, the pilot results and working procedure as of 2015. The introduction was facilitated by the regional GP organisation, who promoted the team in the starting year. The goal of the CandA team was to support GPs and other youth professionals with consultation and guidance to appropriate care, and to provide short-term cognitive behavioural treatment for youth who did not (yet) need referral to primary or specialized mental health care. Hence, the team would provide accessible assessment and care, without raising extra barriers for children, adolescents and their parents. The team has an independent position and is not dependant on or related to a mental health care organization. The tasks of the team are described in **Text box 1**.

**Text box 1 Tasks of the CandA team**

*Tasks of the C and A team at the start in 2015*

1 Supporting youths and parents in exploring and defining mental health problems and care needs

2 Providing consultation to GPs, youth or family counsellors

3 Offering short-term support or treatment for problems related to bullying, anxiety, sleeping problems, divorce, psychosomatic problems, behavioral problems or school absence

*Additional tasks since 2016*

4 Providing advice on referrals to youth mental health care

5 Professionalization and education of family counsellors/social workers

6 Advice to school attendance officers about youths’ obligations to attend school and (medical) exemptions

7 Integrated Early Care and support for 0-to-4 year olds

8 Supporting the municipality in acquisition and contracts with providers of care and support

### Procedure of the CandA team

GPs and other youth professionals can contact the CandA team for consultation by telephone or e-mail. They fill in an application form containing information about the child and the parents/family, the child’s main problems, and any previously received care or support. Anonymous requests for consultation are answered by telephone or e-mail, without application form. The CandA team contacts the parent(s), child or adolescent to discuss the problem, perform an anamnesis and identify the need for care or support. Subsequently, appropriate care is arranged: preventive support or primary care for mild problems, short-term treatment up to 5 sessions with the team’s psychologist, referral to accessible youth public health care or welfare, or referral to specialized mental health care if necessary. Direct referral to specialized mental health services or emergency services takes place in case of an acute crisis. The GP is informed after the intervention of the CandA team by e-mail.

The team additionally initiates meetings to discuss cases and transfer knowledge in order to professionalize youth counsellors. The CandA team was originally located at one office, but since 2017, the team also operates from several GP practices to increase accessibility and visibility to GPs and clients.

### Privacy and information exchange

In order to assure their patients’ privacy, the CandA team is composed of professionals who are bound to professional secrecy. When a professional consults the team, child’s or adolescent’s problems are discussed anonymously and consultations mostly occur by telephone. When a child or parent is referred to the team, the GP or youth professional informs child and parents about the team and asks them to sign an informed consent form to permit the exchange of personal information to the CandA team, such as contact details, demographics, and concise clinical information. This information and the application form are sent to the CandA team via a secure e-mail service. Patient files are documented and only accessible for CandA team members. These files contain basic information about the professional request for consultation, patient age and gender, problem and symptoms, findings and (tentative) diagnosis, and advise or referral provided by the team. Anonymous cases are filed with consultation date, professional and case information. Because of the independent status of the CandA team, patients are not recorded in regular YHC or GPs‘ Electronic Health records.

### Aims of the study

This study describes the results of an evaluation of the implementation of the CandA team. The aim is to investigate the scope and activities of the CandA team in terms of characteristics of clients, their mental health problems and provided advice and referrals. Additionally, stakeholders’, health care providers’, parents’ and youths’ experiences and satisfaction with the CandA team are investigated.

## Methods

### Design

Quantitative and qualitative methods were employed in a mixed-methods evaluation, in a convergent parallel design [33]. The quantitative evaluation included registration data from the CandA team. In the qualitative process evaluation, experiences and satisfaction of users of the CandA team were evaluated by means of online focus groups (OFGs) and a face-to-face focus group (FTF). Qualitative methods followed COREQ guidelines [34].

### Procedure and participants

#### Quantitative evaluation

Between 2015 and 2017, CandA team members registered characteristics and mental health problems of all clients, the type of care provider who consulted the team, provided advice by the team and referrals. The clients’ mental health problems were provided orally or written by the care provider asking for support of the CandA team, and were subsequently classified by the CandA YHC physicians, following the International Classification of Primary Care (ICPC). In the Netherlands, the ICPC is the standard for coding and classifying problems and symptoms in GP practices [35].

#### Qualitative evaluation

Purposive sampling was used to recruit focus group participants. Participants eligible for the OFGs were: 1) children and adolescents who had consulted the team, 2) their parents, and 3) health care professionals such as GPs, YHC physicians and youth welfare counsellors who had consulted the team. Potential participants were invited by the researcher (SV) by e-mail. Children or adolescents and their caregivers (N = 60) were informed about the evaluation study via an information letter provided by a CandA team member at the end of the consultation. All children or caregivers potentially interested to join the OFG signed consent forms. They received further information by e-mail (N = 10). A convenience sample of professionals (N = 18) was recruited. All potential participants were informed that participation was voluntary, anonymous and that they could withdraw from the study at any time, without consequences. Participating children and caregivers received a €25 gift card for their time and effort.

We simultaneously ran three homogenous OFGs in September 2017, for professionals, children and parents, using a web-based online focus group application developed for another study [36]. OFGs were asynchronous: participants could respond at any time and place convenient to them, during a one-week period. Two researchers acted as moderators. The OFGs followed a semi-structured topic guide, based on an analysis of policy documents about the CandA team and explorative interviews, questioning participants’ views about the quality of the CandA team’s services. Participants could anonymously log on to the website to answer questions and to react to other participants’ contributions. During five days, one central theme was discussed each day, namely: organization of the CandA team; quality of care provided by the team; general opinions on privacy and information exchange; the team’s privacy and information exchange; and future of the CandA team. Questions of previous days remained open for responses during the whole week.

To further explore issues raised in the OFGs, an additional FTF with GPs was organized. Both GPs who used the services of the CandA team regularly and GPs who had consulted the team only once or twice, were invited to participate by a YHC staff physician. Potential participants received further information about the FTF by e-mail. Participants were informed that their participation was voluntary. Each participant signed an informed consent form.

The FTF was audio recorded and transcribed verbatim. The transcript was de-identified, assuring confidentiality and anonymity. The moderator (SV) encouraged participants to share their own opinions, with respect for the opinions of other group members. The topics were comparable to the ones in the OFGs, but somewhat adapted: development of the CandA team, the role of GPs in youth mental health care and collaboration with other professionals; aims and composition of the CandA team; reasons for contacting the team; privacy issues and information exchange; and future development of the CandA team.

This study was approved by the Medical Ethics Review Committee of VU University Medical Center, declaring that the Medical Research Involving Human Subjects Act does not apply (2016.367).

### Analyses

Descriptive statistical analyses of the registration data were used to investigate the number and characteristics of clients of the CandA team, their mental health problems, the type of care providers consulting the team, and provided advise and referrals by the team. Chi-squared tests were executed for comparison between the years.

Digital transcripts of the OFGs were used for a combined deductive (based on the topic guides) and inductive analysis. Transcripts of the interviews, OFGs and FTF were coded in ATLAS.ti 7 software and on paper. Two researchers each read the transcripts independently and constructed a thematic coding scheme. Disagreements in coding were discussed until consensus was achieved.

## Results

### Characteristics of the target group

From 1 January 2015 until 31 December 2017, 706 children and adolescents were assessed by the CandA team (Table 1). Mean child age was 10.6 years (range 0–18, SD 4.3), 48% of youths were boys and 38% were girls. The most prevalent problems were ‘other behavioural or mental problems or disorders’ (ICPC codes P22, P23, P29; 41%, e.g. educational, family, or social problems), irritable or angry behaviour (ICPC code P04; 14%), feeling anxious or nervous or anxiety disorder (ICPC codes P01, P74; 10%), overactivity (ICPC code P21; 8%) and feeling down or depressed or depression (ICPC codes P03, P76; 6%). Other problems such as sleeping, eating, language or speech problems, autism, enuresis, or drug use were not or seldom (<2%) recorded.

**Table 1:** Registration data of the CandA team over the years 2015, 2016, 2017.


CandA TEAM CASES	TOTAL	2015	2016	2017	

	N = 706	N = 217	N = 279	N = 210	Chi^2^

**Age:** Mean (SD)	10.6 (4.3) Range 0–18	11.0 (4.0) Range 2–18	10.7 (4.6) Range 0–18	10.2 (4.3) Range 0–17	

Age group 0–12	338 (48%)	83 (38%)	127 (46%)	128 (61%)	

Age group 13–18	217 (31%)	56 (26%)	90 (32%)	71 (34%)	

missing	*150 (21%)*	*77 (36%)*	*62(22%)*	*11 (5%)*	

**Gender**					

Male	338 (48%)	99 (46%)	135 (48%)	104 (50%)	

Female	270 (38%)	89 (41%)	87 (31%)	94 (45%)	

missing	*98 (14%)*	*29 (13%)*	*57 (20%)*	*12 (6%)*	

**Problems/symptoms (ICPC code)**1					

Other behavioural/mental problems (P22, P23, P29)	287 (41%)	69 (32%)	106 (38%)	112 (54%)	14.54**

Irritable/angry (P04)	95 (14%)	26 (12%)	44 (16%)	25 (12%)	1.73

Anxious/nervous (P1, P74)	68 (10%)	30 (14%)	19 (7%)	19 (9%)	15.48***

Overactivity (P21)	53 (8%)	21 (10%)	16 (6%)	16 (8%)	6.37*

Down/depressed (P03, P76)	44 (6%)	9 (4%)	18 (6%)	17 (8%)	1.12

missing	*166 (24%)*	*81 (37%)*	*48 (17%)*	*37 (18%)*	

**Referrers**					

General Practitioner	339 (48%)	121 (56%)	115 (41%)	103 (49%)	10.47**

Youth Welfare	139 (20%)	42 (19%)	65 (23%)	32 (15%)	4.94

YMH Practice Nurse	35 (5%)	7 (3%)	6 (2%)	22 (10%)	19.62***

Preventive YHC	76 (11%)	19 (8%)	28 (10%)	29 (14%)	3.09

other	116 (16%)	28 (13%)	65 (23%)	23 (11%)	16.14***

missing	1 (0.1%)	0	0	1 (0.5%)	

**Request for**					

Consultation	398 (56%)	102 (47%)	179 (64%)	117 (56%)	5.20

Exploring problem and advise	259 (37%)	75 (35%)	95 (34%)	89 (42%)	4.17

Short-term treatment	4 (1%)	2 (1%)	1 (0.5%)	2 (1%)	2.90

missing	*44 (6%)*	*38 (18%)*	*4 (1%)*	*2 (1%)*	

**Resulting in**					

Advise	446 (63%)	106 (49%)	199 (71%)	141 (67%)	9.55^**^

Direct referral	129 (18%)	41 (19%)	50 (18%)	38 (18%)	1.79

Short- term treatment	33 (5%)	16 (7%)	10 (4%)	7 (3%)	7.93^*^

Monitoring/watchful waiting	15 (2%)	7 (3%)	6 (2%)	2 (1%)	3.61

missing	*82 (12%)*	*47 (22%)*	*14 (5%)*	*21 (10%)*	

**Referral to**					

Primary MHC	58 (8%)	19 (9%)	17 (6%)	22 (11%)	6.97*

Specialized MHC	116 (16%)	35 (16%)	46 (17%)	35 (17%)	2.87

Youth welfare and care	31 (4%)	7 (3%)	9 (3%)	15 (7%)	5.99*

Front line support	107 (15%)	19 (9%)	44 (16%)	44 (21%)	9.27*

Other	128 (18%)	33 (15%)	64 (23%)	31 (15%)	2.38

No referral	108 (15%)	23 (11%)	70 (25%)	15 (7%)	22.71***

missing	158 (22%)	80 (37%)	29 (10%)	43 (23%)	


^1^ The five most prevalent categories of ICPC problems/symptoms. Other problems were prevalent in <2%. For some cases, more than one code was registered, hence percentages may add up to >100%. * p-value < .05; **p-value < .01; *** p-value < .001.

CandA team services were mostly used by GPs (48%), youth welfare counsellors (20%), preventive youth health care physicians or nurses (11%) or physician’s assistant nurses working with GPs (5%). Other professionals also used the team’s services (16%), for example psychologists, school attendance officers or educationalists. In total, 69 GPs from 37 GP-practices used the CandA team services (46% of all GPs in the region, 67% of all GP-practices in the region, data not in table 1, https://www.rhogo.nl).

More than half of the contacts with the CandA team were consultations (56%). In 37% of the cases, the CandA team helped children or adolescents and their parents to explore the problem and identify their care needs. In 63% of the cases, the team advised other professionals about care or referral, in 18% they referred directly, 5% of youth received short-term treatment from the CandA team.

Of the 706 youths who were assessed by the CandA team, 8% were referred to primary mental health care, 16% to specialized mental health care, 15% to front line support such as social work or educational support and 4% to youth welfare. Another 18% were referred to other services, and 15% were not referred at all. During the three years of the study, referrals to primary mental health care, youth welfare or front line support increased, referrals rates to specialized mental health care remained the same.

### Youths’, parents’ and professionals’ perspectives and experiences concerning the CandA team

Table 2 provides an overview of topics discussed in the four focus groups. For each topic and each group of participants, the evaluation (positive or negative) they mentioned is described, and whether any suggestions for improvement were mentioned.

**Table 2:** Evaluation of the CandA team expressed in focus groups with GPs (N = 5), youth professionals (N = 7), parents (N = 4), and youths (N = 4).


	POSITIVE OPINION				NEGATIVE OPINION				SUGGESTION FOR IMPROVEMENT			
		
	GP	Pr	Pa	Y	GP	Pr	Pa	Y	GP	Pr	Pa	Y

**General satisfaction with the CandA team**												

Execution of CandA tasks and working methods	X	X	X	X								

Being treated with respect by CandA team	X	X	X	X								

**Perceived aims and tasks of the CandA team**												

Supporting youths parents in exploring and defining their mental health problems and care needs	X		X	X			X					

Supporting GPs and youth professionals with consultation and guidance to appropriate care	X	X	X	X	X				X	X		

Offering short-term support or treatment to youths parents	X	X							X	X		

Providing care counselling during waiting time for other type of care							X		X	X	X	

Cooperation with schools					X				X			

**Composition and accessibility of the CandA team**												

The CandA team is a multidisciplinary expert team	X	X				X			X	X		

Workload of CandA team members	X					X			X	X		

Accessibility of CandA team for professionals	X	X			X	X			X	X		

Accessibility of CandA team for youths and parents							X	X	X	X		X

Short waiting time for support of CandA team	X	X		X	X				X		X	

**Information exchange and privacy**												

Ensuring privacy by professional secrecy codes		X							X			

Ways of information exchange between professionals		X				X			X			

Ensuring privacy in interprofessional information exchange by using an informed consent form		X	X									

Opinions on who should (not) be informed or have access to information					X							


GP: general practitioners; Pr: professionals; Pa: parents; Y: youths. X denotes whether an aspect is mentioned in the respective focus group and which evaluation (positive or negative) is given to this aspect, or whether any suggestion for improvement is mentioned.

#### General satisfaction with the CandA team

All participant groups were satisfied with the team’s working methods, knowledge, expertise and service delivery. Particularly the fact that professionals, youths and parents were treated respectfully was highly appreciated.

“It was a pity that I could only come once, because the doctor was really nice.” (youth)

“We felt heard and understood. They addressed our problems.” (parent)

“Our problems were acknowledged before I even told exactly what was going on. My child and I felt we were taken seriously.” (parent)

#### Aims and tasks of the CandA team

Participating GPs, youth professionals, youths and parents were mainly positive about the aims of the CandA team and the way the team performed its tasks. They were particularly positive about the way in which the CandA team provides GPs and youth professionals with consultation and guidance to appropriate care.

“The team is an indispensable link. It is very good that young people can talk to them, when they experience a threshold for using mental health care.” (parent)

“It works for me, because they act fast and easy, and they always report back what they did.” (GP)

“I am very enthusiastic, really good that the team exists! I used to refer to a care organization and had no idea whether that would be adequate. Now I know for sure when a referral is adequate.” (GP)

Parents and youths were positive about being asked the right questions by CandA team members. Some parents mentioned that the CandA team could play a role in bridging the long waiting time for mental health care.

“It is a pity that after we consulted them and they gave referral advise, our son was eager to get help, but had to wait for months to see a psychologist.” (parent)

GPs’ and youth professionals’ main suggestions for improvement concerned whether and how the CandA team could contribute to improved prevention, early recognition of psychosocial problems and stronger interprofessional collaboration. Many problems of youths consulting GPs are related to school (e.g. learning problems, attention and hyperactivity problems, or bullying) or to the family (e.g. problems related to divorce). GPs indicated that they often refer these children to specialized mental health care, but that they doubt the adequacy of this path. GPs suggested to establish a direct link between schools and the CandA team.

“School is important, they see and detect problems. It is a natural way to go from school to the CandA team members, they [youths] do not have to come and see us.” (GP)

#### Composition and accessibility of the CandA team

Some youth professionals and GPs were positive about having access to an interdisciplinary expert team and to be able to discuss cases with a physician or psychologist.

“They have really good expertise and knowledge of the network.” (professional)

“They know what they are talking about. It gives me confidence in their advice.” (GP)

Some professionals were worried that the current formation of the CandA team might not be sufficient to cope with the workload. They therefore suggested an expansion of the team. On the other hand, some GPs thought that the CandA team’s strength is its small size.

All participant groups were negative about the accessibility of the team, as the team was not accessible during school holidays and worked from only one location. Some youths and parents indicated they had to travel too far for a face-to-face appointment. Youths suggested that consultations could take place in school or closer to their home. Some professionals indicated that they would prefer the possibility of telephone consultations or face-to-face meetings with the CandA team. On the other hand, GPs who had the team operating from their own practice – a pilot in two specific municipalities – were very positive about the accessibility of the team and thought it improved the accessibility for youth and parents. Youth professionals, GPs and youths expressed mainly positive opinions about the short waiting time for receiving support from the CandA team.

#### Information exchange and privacy

Youth professionals thought privacy was assured, because the CandA team members are bound to professional secrecy. GPs expressed doubts about the safety of information exchange via e-mail, and mentioned that this could jeopardize future referrals to the team.

“If I had to refer a child to the CandA team at this moment, I might not do it, because I cannot e-mail safely. That’s an urgent problem.” (GP)

Parents thought their privacy in interprofessional information exchange was assured by the use of an informed consent form.

## Discussion

According to GPs, youth professionals, adolescents, and parents, the CandA team does what it was set out to do and does it well: supporting professionals in the identification of child and adolescent mental health problems, supporting youth and parents, and referring to appropriate care. All stakeholders were positive about the CandA team, particularly about the team’s expertise and service delivery. Stakeholders were particularly satisfied with the fact that professionals and clients were treated respectfully and were taken seriously. Parents and professionals felt the clients’ privacy was assured, because of the informed consent form and codes of conduct of the involved professionals. However, professionals expressed concerns about information exchange via (unprotected) e-mail. In this respect, the independent status of the CandA team has pros and cons. Patient information is not recorded in regular YHC or GPs’ electronic health records. This could be a barrier to coordinated care. These concerns are justified and should be addressed. Although the CandA team members are individually bound to professional secrecy, the CandA team in itself is not, and privacy is therefore not officially assured [37].

Other suggestions for improvement concerned the accessibility of the team in terms of time and location, cooperation with schools and bridging the long waiting period for specialized mental health care.

The objectives of the CandA team are comparable to those of other Dutch initiatives after the introduction of the Youth Act in 2015 [38]. However, the CandA team is unique because of the central role of YHC physicians and the team’s independent position. The CandA team members have a good overview of the social care and support services in the community, enabling them to advise about or refer to appropriate care. This is essential for interagency cooperation: ‘consider developing a greater understanding of other services in the field, adequate channels of communication with them and positive individual relationships’ [39].

The CandA team supported more than 700 children and adolescents in three years. The team did this directly, by listening to youths and parents and clarifying their problems, or indirectly, by providing consultation to GPs and other youth professionals. The CandA team’s services were used mainly by GPs, but also by youth welfare counsellors, YHC physicians, physician assistant nurses and other youth professionals. After consultation or advise, the team referred some children or adolescents to other services.

The number of cases supported by the CandA team, stayed more or less steady in the first and third year reported, with an increase in the middle year. More than half of the GPs in the region did not use the team, hence a more active promotion of the team’s services and added value may be necessary. It is also possible that GPs who were supported by the team, learned from their expertise and know better how to act or refer directly in similar cases in the future.

Unspecified mental health problems in the category ‘other problems’ were recorded most frequently in the CandA team population, indicating that GPs, other professionals or parents seek support from the CandA team for less clear and/or difficult to interpret mental health problems. GPs in the focus groups confirmed that they particularly consulted the CandA team when children or adolescents had vague problems, and when it was unclear whether the origin was psychiatric, psychosocial or systemic. Hence, the CandA team has extra value for unclear and not yet diagnosed problems. This is in line with a study in which the highest prevalence of psychological symptoms and diagnoses within general practices with extra mental health support, was found within the category ‘other concerns about the child’s behaviour’ [4].

Most Dutch local or regional initiatives to support GPs in the management of youths with mental health problems, were mainly implemented to decrease the number of youths being referred to specialized mental health services and thereby reduce the costs of mental health care [28]. These aims were based on the Dutch Youth Act from 2015. An evaluation of this Act in 2018 concluded that the intended decrease in referrals to specialized mental health services had not yet occurred [40]. Our results confirm this conclusion. Integrated mental healthcare approaches within general practices may lead to an increase in detected psychosocial problems and therefore a decrease of referrals may not be expected [4]. Our study did not evaluate whether there was an increase in the detection of mental health problems after the implementation of the CandA team. This may be recommended for a future study.

Reduction of referrals to specialized mental health care should not be the main goal. Some children and adolescents show such serious or complex problems, that specialized care is warranted. Instead of focusing on reducing the number of referrals to specialized mental health care, it is more important to focus on referring youth to the most appropriate type of care, which optimally contributes to their wellbeing. Good quality triage between youths who can be adequately supported by primary mental health care or community support and the ones who need specialized care, is essential [19,20].

All schools in the Netherlands have a YHC physician appointed to them, but their work at schools is very limited [41]. Schools can play an important role in detecting mental health problems related to learning disabilities, attention and hyperactivity problems, bullying or family problems [42]. In our study, GPs hardly ever consulted the CandA team for children or adolescents with learning disabilities, while previous studies show that specific learning problems are a prevalent diagnosis in general practice [4]. GPs in our study might have assumed that these problems are already addressed by (school)professionals, and/or they directly referred these children to (specialized) mental health care. If the latter is the case, the burden of these diagnoses on GPs may still be high. Furthermore, literature shows that many children or adolescents with mental health problems do not consult their GP [43,44,45]. A stronger collaboration between approachable experts like the ones in the CandA team and schools might improve early identification and treatment of children and adolescents with mental health problems.

### Strengths and limitations

To our knowledge, this is one of the few studies to evaluate a team that connected primary care, public health care and mental health care, to support professionals with the early identification and appropriate management of child and adolescent mental health problems. The strength of this evaluation study is the combination of quantitative and qualitative research methods, providing data from many perspectives.

The study has two main limitations. First, there were low response rates for all focus groups. Only four youths and four parents participated. This could limit the validity and generalisability of our findings. However, such response rates are not unusual in qualitative evaluation studies. Furthermore, focus groups are primarily meant to explore the views and opinions of small groups of people, instead of generalizing findings [46]. Although we intended to recruit both GPs who contacted the CandA team regularly and GPs who seldom or never did, we ended up with only the first group. This may have caused a positive bias and higher satisfaction. However, participating GPs thought that their colleagues’ reasons for not contacting the CandA team were related to unfamiliarity rather than dissatisfaction.

Second, many registration data from 2015 are missing, particularly data about children’s age group, mental health problems and referrals. Many consultations were anonymous and only information relevant to cases was registered. In addition, data from 2015 were registered at the time the study had not officially started yet. When the study started in 2016, data from 2016 and 2017 were registered more strictly in collaboration with the researchers, hence registration of all relevant variables was safeguarded.

## Conclusion

A multidisciplinary consultation and advise expert team on child and adolescent mental health problems (CandA team), in which PHC physicians and a psychologist support GPs, youth health or welfare professionals, parents and youth with the management of mental health problems, is a highly accepted and appropriate form of integrated care for the assessment of child and adolescent mental health problems in the community. The expertise of the team members, their independent position in the community and the way the team operates, are important success factors. A few points for improvement were raised and some privacy issues should be addressed.

The CandA team has already taken on a central role in strengthening interagency collaboration in the region and has potential to grow. Quantitative evaluation is needed to further investigate the effects of the CandA team on the identification and treatment of mental health problems, and referrals to mental health care.

## References

[1] World Health Organisation. World mental health report: transforming mental health for all. Geneva: WHO; 2022. Contract No.: ISBN: 9789240049338.

[2] Zeijl E, Crone M, Wiefferink K, Keuzenkamp S, Reijneveld M. Kinderen in Nederland [Children in the Netherlands]. Leiden; 2005.

[3] Boer M, van Dorsselaer S, de Looze M, de Roos S, Brons H, van den Eijnden R, Monshouwer K, Huijnk W, ter Bogt T, Vollebergh W, Stevens G. HBSC 2021 Gezondheid en welzijn van jongeren in Nederland [Health Behaviour of School-aged Children 2021. *Health and wellbeing of youth in the Netherlands*]; 2022. Utrecht: Universiteit Utrecht.

[4] Verhaak PF, van Dijk M, Walstock D, Zwaanswijk M. A new approach to child mental healthcare within general practice. BMC Fam Pract. 2015; 16: 132. DOI: 10.1186/s12875-015-0354-2 26452756 PMC4600203

[5] Zwaanswijk M, van Dijk CE, Verheij RA. Child and adolescent mental health care in Dutch general practice: time trend analyses. BMC Fam Pract. 2011; 12: 133. DOI: 10.1186/1471-2296-12-133 22133283 PMC3267656

[6] Bot M, de Leeuw den Bouter BJ, Adriaanse MC. Prevalence of psychosocial problems in Dutch children aged 8–12 years and its association with risk factors and quality of life. Epidemiol Psychiatr Sci. 2011; 20(4): 357–65. DOI: 10.1017/S2045796011000540 22201213

[7] Tick NT, van der Ende J, Verhulst FC. Twenty-year trends in emotional and behavioral problems in Dutch children in a changing society. Acta Psychiatr Scand. 2007; 116(6): 473–82. DOI: 10.1111/j.1600-0447.2007.01068.x 17997726

[8] Collishaw S. Annual research review: Secular trends in child and adolescent mental health. J Child Psychol Psychiatry. 2015; 56(3): 370–93. DOI: 10.1111/jcpp.12372 25496340

[9] Statistics Netherlands. Jeugdhulp 2015 [Youth care 2015]; 2016. DOI: 10.1787/int_trade-v2015-4-24-en

[10] Statistics Netherlands. Jeugdhulp 2022 [Youth care 2022]; 2023.

[11] Lempinen L, Luntamo T, Sourander A. Changes in mental health service use among 8-year-old children: a 24-year time-trend study. Eur Child Adolesc Psychiatry. 2019; 28(4): 521–30. DOI: 10.1007/s00787-018-1218-9 30220075

[12] Ford T, Hamilton H, Goodman R, Meltzer H. Service Contacts Among the Children Participating in the British Child and Adolescent Mental Health Surveys. Child Adolesc Ment Health. 2005; 10(1): 2–9. DOI: 10.1111/j.1475-3588.2005.00108.x 32806819

[13] Zijlmans J, Tieskens JM, van Oers HA, Alrouh H, Luijten MAJ, de Groot R, et al. The effects of COVID-19 on child mental and social health: biannual assessments up to April 2022 in a clinical and two general population samples. medRxiv. 2022; 2022.09.08.22279670. DOI: 10.1002/jcv2.12150 PMC10519731 37753155

[14] Kessler RC, Amminger GP, Aguilar-Gaxiola S, Alonso J, Lee S, Ustün TB. Age of onset of mental disorders: a review of recent literature. Curr Opin Psychiatry. 2007; 20(4): 359–64. DOI: 10.1097/YCO.0b013e32816ebc8c 17551351 PMC1925038

[15] Sheldrick RC, Merchant S, Perrin EC. Identification of developmental-behavioral problems in primary care: a systematic review. Pediatrics. 2011; 128(2): 356–63. DOI: 10.1542/peds.2010-3261 21727101

[16] Ormel J, Raven D, van Oort F, Hartman CA, Reijneveld SA, Veenstra R, et al. Mental health in Dutch adolescents: a TRAILS report on prevalence, severity, age of onset, continuity and co-morbidity of DSM disorders. Psychol Med. 2015; 45(2): 345–60. DOI: 10.1017/S0033291714001469 25066533

[17] Netherlands Youth Institute. Reform of the Dutch system for child and youth care, 4 years later. Utrecht: Netherlands Youth Institute; 2019.

[18] Siderius EJ, Carmiggelt B, Rijn CS, Heerkens YF. Preventive Child Health Care within the Framework of the Dutch Health Care System. J Pediatr. 2016; 177s: S138–s41. DOI: 10.1016/j.jpeds.2016.04.050 27666262

[19] Bezem J, Kocken PL, Kamphuis M, Theunissen MHC, Buitendijk SE, Numans ME. Triage in preventive child healthcare: a prospective cohort study of care use and referral rates for children at risk. BMJ Open. 2017; 7(10): e016423. DOI: 10.1136/bmjopen-2017-016423 PMC5665215 29084789

[20] Wissow LS, van Ginneken N, Chandna J, Rahman A. Integrating Children’s Mental Health into Primary Care. Pediatr Clin North Am. 2016; 63(1): 97–113. DOI: 10.1016/j.pcl.2015.08.005 26613691 PMC4663456

[21] Door de huisarts geregistreerde contacten; leeftijd en geslacht, 2006–2018 [General Practitioners registrated consultations; age and gender, 2006–2018] [Internet]. Statistics Netherlands. 2021 [cited April, 2023]. Available from: https://opendata.cbs.nl/statline/#/CBS/nl/dataset/80191ned/table?ts=1552646057612.

[22] Vanneste YTM, Lanting CI, Detmar SB. The Preventive Child and Youth Healthcare Service in the Netherlands: The State of the Art and Challenges Ahead. International Journal of Environmental Research and Public Health. 2022; 19(14): 8736. DOI: 10.3390/ijerph19148736 35886585 PMC9320981

[23] Jambroes M, Lamkaddem M, Stronks K, Essink-Bot ML. Enumerating the preventive youth health care workforce: Size, composition and regional variation in the Netherlands. Health Policy. 2015; 119(12): 1557–64. DOI: 10.1016/j.healthpol.2015.08.002 26358246

[24] Wieske RC, Nijnuis MG, Carmiggelt BC, Wagenaar-Fischer MM, Boere-Boonekamp MM. Preventive youth health care in 11 European countries: an exploratory analysis. Int J Public Health. 2012; 57(3): 637–41. DOI: 10.1007/s00038-011-0305-1 21956621 PMC3359454

[25] Zorgwijzer. Healthcare in the Netherlands 2024. Available from: https://www.zorgwijzer.nl/faq/healthcare-netherlands.

[26] Netherlands Youth Institute. Introduction to Dutch youth policy: Netherlands Youth Institute; 2021. Available from: https://www.nji.nl/english/introduction-dutch-youth-policy.

[27] Theunissen MHC, Dijkshoorn JJ, Klein Velderman M. Specialistische ondersteuning in de basiszorg voor jeugd: verbindingen maken in het sociale domein. Tijdschrift voor gezondheidswetenschappen. 2018; 96(8): 354–60. DOI: 10.1007/s12508-018-0206-2

[28] Otten E, Zwaanswijk M, Koopman I. Specialistische ondersteuner huisartsenzorg jeugd-GGZ, SOH-JGGZ [Specialist support in general practice youth mental health]. Bijblijven [Keeping Up in mental health]. 2018; 16(8): 1–20. DOI: 10.1007/s12414-018-0347-x

[29] Theunissen MHC, Dijkshoorn JJ, Klein Velderman M. Specialistische ondersteuning in de basiszorg voor de jeugd: verbinding maken in het sociale domein. Tijdschr gezondheidswet; 2018. DOI: 10.1007/s12508-018-0206-2

[30] Koning NR, van der Schriek LMM, van der Kooij MJ, Büchner FL, de Wilde JA, Numans ME, Crone MR. Collaboration between general practitioners and preventive youth health physicians: room for improvement. Ned Tijdschr Geneeskd. 2018; 162. 30040274

[31] Derksen-Lubsen G, Jambroes M, Essink-Bot ML. Healthcare for teenagers: are we working together? Ned Tijdschr Geneeskd. 2016; 160: D783. 27581869

[32] Jambroes M, Essink-Bot ML, Plochg T, Zaadstra B, Stronks K. Public health services in the Netherlands: 10 core functions and a new definition. Ned Tijdschr Geneeskd. 2013; 157: A6195.

[33] Schoonenboom J, Johnson RB. How to Construct a Mixed Methods Research Design. Kolner Z Soz Sozpsychol. 2017; 69(Suppl 2): 107–31. DOI: 10.1007/s11577-017-0454-1 28989188 PMC5602001

[34] Tong A, Sainsbury P, Craig J. Consolidated criteria for reporting qualitative research (COREQ): a 32-item checklist for interviews and focus groups. International Journal for Quality in Health Care. 2007; 19(6): 349–57. DOI: 10.1093/intqhc/mzm042 17872937

[35] Lamberts H, Woods M, Hofmans-Okkes I. The international classification of primary care in the European Community. Oxford: Oxford University Press; 1993.

[36] Tates K, Zwaanswijk M, Otten R, van Dulmen S, Hoogerbrugge PM, Kamps WA, Bensing JM. Online focus groups as a tool to collect data in hard-to-include populations: examples from paediatric oncology. BMC Med Res Methodol. 2009; 9: 15. DOI: 10.1186/1471-2288-9-15 19257883 PMC2653071

[37] Dörenberg V, Vandenbussche SV, Hoogsteder MHH. Tussen huisarts, jeugdarts en gemeente in de regio Gooi en Vechtstreek. Een juridische beschouwing over het ‘Consultatie en Adviesteam jeugd-GGZ’ [Between General Practitioner, Youth Health physician and municipality in the Gooi and Vechtstreeks region. A legal discussion about the Consultation and Advise team youth mental health]. Amsterdam UMC, Health DoPaO; 2019 mei 2019.

[38] Raaijmakers F, Klein Velderman M. Specialistische ondersteuning in de basiszorg voor jeugd. TSG – Tijdschrift voor gezondheidswetenschappen. 2020; 98(3): 114–7. DOI: 10.1007/s12508-020-00270-y

[39] Cooper M, Evans Y, Pybis J. Interagency collaboration in children and young people’s mental health: a systematic review of outcomes, facilitating factors and inhibiting factors. Child Care Health Dev. 2016; 42(3): 325–42. DOI: 10.1111/cch.12322 26860960

[40] Friele RD, Bruning MR, Bastiaanssen ILW, De Boer R, Bucx AJEH, De Groot JF, Pehlivan T, Rutjes L, Sondeijker F, Van Yperen TA, Hageraats R. Eerste evaluatie Jeugdwet: na de transitie nu de transformatie [First evaluation Youth Act: After the transition, yet the transformation]; 2018.

[41] Vanneste Y, Rots C, van de Goor I, Feron F. Medische Advisering Ziekgemelde Leerling door de jeugdarts (M@ZL) [Medical Advice for Sick-reported Students (MASS) in secondary school by the youth health care physician: development of an intervention]. Tijdschrift voor gezondheidswetenschappen. 2012; 90(7): 412–9. DOI: 10.1007/s12508-012-0145-2

[42] Loades ME, Mastroyannopoulou K. Teachers’ Recognition of Children’s Mental Health Problems. Child Adolesc Ment Health. 2010; 15(3): 150–6. DOI: 10.1111/j.1475-3588.2009.00551.x 32847228

[43] Zwaanswijk M, van der Ende J, Verhaak PF, Bensing JM, Verhulst FC. Help-seeking for child psychopathology: pathways to informal and professional services in the Netherlands. J Am Acad Child Adolesc Psychiatry. 2005; 44(12): 1292–300. DOI: 10.1097/01.chi.0000181038.98712.c6 16292122

[44] Zwaanswijk M, Verhaak PF, van der Ende J, Bensing JM, Verhulst FC. Consultation for and identification of child and adolescent psychological problems in Dutch general practice. Fam Pract. 2005; 22(5): 498–506. DOI: 10.1093/fampra/cmi045 15964870

[45] Ford T, Goodman R, Meltzer H. Service use over 18 months among a nationally representative sample of British children with psychiatric disorder. Clinical Child Psychology and Psychiatry. 2003; 8: 37–51. DOI: 10.1177/1359104503008001006

[46] Stewart K, Williams M. Researching online populations: the use of online focus groups for social research. Qualitative Research. 2005; 5(4): 395–416. DOI: 10.1177/1468794105056916

